# Measurements of Corneal Thickness in Eyes with Pseudoexfoliation Syndrome: Comparative Study of Different Image Processing Protocols

**DOI:** 10.1155/2017/4315238

**Published:** 2017-09-07

**Authors:** Katarzyna Krysik, Dariusz Dobrowolski, Katarzyna Polanowska, Anita Lyssek-Boron, Edward A. Wylegala

**Affiliations:** ^1^Department of Ophthalmology with Pediatric Unit, St. Barbara Hospital, Trauma Center, Medykow Square 1, 41-200 Sosnowiec, Poland; ^2^Chair and Clinical Department of Ophthalmology, School of Medicine with the Division of Dentistry in Zabrze, Medical University of Silesia in Katowice, Panewnicka 65 st., 40-760 Katowice, Poland; ^3^Department of Ophthalmology, District Railway Hospital, Panewnicka 65 st., 40-760 Katowice, Poland; ^4^Hebei Provincial Eye Hospital, Xingtai, China

## Abstract

**Purpose:**

Comparative analysis of central and peripheral corneal thickness in PEX patients using three different imaging systems: Pentacam-Scheimpflug device, time-domain optical coherence tomography (OCT) Visante, and swept-source OCT Casia.

**Materials and Methods:**

128 eyes of 80 patients with diagnosed PEX were examined and compared with 112 normal, non-PEX eyes of 72 cataract patients. The study parameters included 5 measured zones: central and 4 peripheral (superior, inferior, nasal, and temporal).

**Results:**

The mean CCT in eyes with PEX syndrome measured with all three instruments was thicker than that in normal eyes. Corneal thickness measurements in the PEX group were statistically significantly different between Pentacam and OCT Casia: central corneal thickness (*p* = 0.04), inferior corneal zone (*p* = 0.01), and nasal and temporal corneal zones (*p* < 0.01). Between Pentacam and OCT Visante inferior, nasal and temporal corneal zones were statistically significantly different (*p* < 0.01). Between OCT Casia and OCT Visante, there were no statistically significant differences in measured parameters values.

**Conclusion:**

The central corneal thickness in eyes with PEX syndrome measured with three different independent methods is higher than that in the non-PEX group, and despite variable peripheral corneal thickness, this one parameter is still crucial in intraocular pressure measurements.

## 1. Introduction

Pseudoexfoliation (PEX) syndrome is a common age-related generalized disease that is characterised by the abnormal production and turnover of extracellular polymorphic fibrillar material, which accumulates at the place of production and does not undergo degradation. The intraocular cells involved in this production include nonpigmented ciliary epithelial cells, posterior pigment epithelial cells of the iris, pre-equatorial lens capsule epithelial cells, corneal endothelium, trabecular cells, endothelial cells of blood vessels and its adventitia, muscular cells, and ganglion cells of the retina. PEX occurs bilaterally, but its manifestation is usually asymmetric [1–3]. PEX affects up to 30% of people older than 60 worldwide [2, 4]. It mainly involves the anterior segment of the eye [1, 5], where its ocular manifestations include phacodonesis, lens subluxation, melanin dispersion, insufficient mydriasis, blood-aqueous barrier dysfunction, anterior chamber hypoxia, posterior synechiae, and corneal endothelial decompensation [1, 2, 5, 6]. PEX is one of the most common causes of ocular hypertension and glaucoma [1, 2, 5, 7].

Quantitatively reduced and morphologically altered corneal endothelium in PEX eyes may lead to a distinct type of keratopathy which diffusely involves the entire cornea. Even moderate rises in intraocular pressure (IOP) or surgical manipulations in the anterior chamber may trigger corneal oedema and decompensation. Reduction of the IOP often leads to clearing of the cornea [5]. However, in advanced stages of PEX-related kerato/endotheliopathy, the potential reversing endothelial decompensation may be limited. This leads to decreased visual acuity, and it is often accompanied by ocular pain; finally, corneal transplantation is required to treat this condition [2, 5].

In addition to intraocular, PEX patients also present with different systemic manifestations that are mainly associated with cardiovascular and cerebrovascular morbidities (myocardial infarction or stroke, arterial hypertension, transient ischemic attacks, aneurysms of the abdominal aorta, thromboses, embolisms, haemorrhages, cerebral ischemia, Alzheimer's disease, and sensorineural hearing loss) [1–3, 5].

The observation and examination of the anterior segment of the eye by using slit-lamp biomicroscopy is subjective. The imaging and evaluation of anterior segment structures demands the application of new, objective, noninvasive imaging technologies which provide quantitative and qualitative assessments of all structures. Different devices are available for measuring the corneal thickness. Ultrasound pachymetry was considered the gold standard for pachymetry. However, this technique is limited, because only specific points can be measured, and not the global pachymetry; furthermore, it requires aseptic precautions and local anaesthesia [8, 9]. Other noninvasive techniques that can be used to determine the global corneal thickness were introduced, including optical coherence tomography (OCT), ultrasonic biomicroscopy, scanning slit topography, scanning peripheral anterior chamber depth analyser, and Pentacam-Scheimpflug imaging [8–13].

The aim of this study was to comparatively analyse the central and peripheral corneal thickness in PEX patients using three different imaging systems: Pentacam-Scheimpflug camera, Visante time-domain OCT, and Casia swept-source OCT.

## 2. Patients and Methods

This study was performed at the Ophthalmology Department of Saint Barbara Hospital, Trauma Centre, Sosnowiec, Poland. All subjects with diagnosed ophthalmic symptoms of PEX were recruited from among the department's cataract patients. Informed consent was obtained from all participants. All patients underwent a complete ophthalmic examination, including best-corrected distance visual acuity, IOP measurement by Goldmann applanation tonometry, slit-lamp biomicroscopy, and fundus examination with a dilated pupil. The inclusion criteria were the presence of pseudoexfoliative material on the anterior lens capsule, pupil margin, or both.

The exclusion criteria were other ophthalmic or systemic conditions which could influence corneal thickness measurements, such as corneal pathology (dystrophies and degenerations, scars, and status postcorneal refractive surgery), previous ocular surgeries or ocular trauma, glaucoma, ocular hypertension, uveitis, diabetes, systemic diseases with ocular manifestation (collagen, skin, or mucous membrane diseases), eyes with refractive errors (±4.0 spherical diopters and ±2.0 cylindrical diopters), and usage of topical medication which might affect the corneal condition (especially medications with preservatives).

All 128 phakic eyes of 80 patients (48 females and 32 males) with diagnosed PEX were examined and compared with 112 normal, non-PEX eyes of 72 cataract patients (40 females and 32 males). Patients from both groups were recruited from cataract patients operated on between October 1, 2016 and February 28, 2017.

All corneal thickness measurements were obtained by one operator using the three imaging systems mentioned above. The study parameters were delivered from anterior chamber images, which were processed for five zones: one central and four peripheral (superior, inferior, nasal, and temporal).

## 3. Instruments

The Pentacam-Scheimpflug imaging system (Pentacam HR, Oculus, Wetzlar, Germany) captures 100 slit images with a slit depth of 14.0 mm in 2 s by rotating along the optical axis from 0° to 360°. Its digital camera and slit illumination system (475 nm monochromatic light) rotate automatically around the corneal apex to capture cross-sectional images of the anterior eye. A fit zone diameter of 8 mm was applied [9–14].

Swept-source OCT (Casia SS-1000, Tomey, Nagoya, Japan) is a swept-source anterior segment OCT that uses a wavelength of 1310 nm and performs measurements with a speed of 30,000 axial scans per second. In the corneal map mode, each 3D image consists of 16 B-scans and 512 A-lines, and in the anterior segment mode, each 3D scan contains 128 B-scans and 512 A-scans. The total scan duration is 0.3 s for the measurement of the corneal thickness and corneal topography. A fit zone diameter of 8 mm was applied [11, 14–18].

Time-domain OCT (Visante OCT, Carl Zeiss Meditec, Inc., Dublin, California, USA) uses a 1310 nm superluminescent diode source for imaging, which operates at a speed of 4000 axial scans per second. The image acquisition system provides a video image of the examined zone and stores the last 7 images at a rate of 8 frames per second. It generates a pachymetry map with concentric circles with diameters of 2, 5, 7, and 10 mm. A fit zone diameter of 7 mm was applied [9, 13, 15, 16].

Axial resolution, offered by listed devices, is as follows: Pentacam-Scheimpflug camera—10 *μ*m, Visante OCT—18 *μ*m, and Casia OCT—10 *μ*m.

Statistical analysis was performed using Statistica versus 13.1 computer software (StatSoft, USA). The results are presented as mean ± standard deviation. In a Bland-Altman plot, the difference between the measurements with different methods is plotted against their mean. The 95% limits of agreement (mean difference ± 1.96 standard deviation) give the distance between the measurements by the methods with 95% confidence. The Bland-Altman plot also shows the proportional bias in the measurements, which is the relationship of the difference between the measurements and the true value. The parameter values were compared between the normal and the PEX groups by using Student's *t*-test or the Mann–Whitney *U* test. For normal and near-normal distributions, a variance analysis was performed using ANOVA test, and then, the homogeneity of variance was determined using Bartlett's test. A *p* value less than 0.05 was considered statistically significant.

## 4. Results

The mean age of the study group was 73 ± 7.8 years (range: 49–88 years) and that of the control group was 69 ± 9.3 years (range: 45–84 years). There was no statistically significant difference with respect to gender and age between both groups (*p* > 0.05). The mean measurements of the corneal thickness in five zones and its standard deviation in the PEX group and the control non-PEX group are shown in Tables 1 and 2, respectively.

Table 3 shows the differences in pachymetry values (*p* value) between the study and the control groups. A statistically significant difference was found for only two parameters: temporal corneal zone—Pentacam measurement and central corneal thickness (CCT)—Visante measurement.

The corneal thickness measurements in the PEX group were statistically significantly different between Pentacam and Casia: CCT (*p* = 0.04), inferior corneal zone (*p* = 0.01), and nasal and temporal corneal zones (*p* < 0.01). Between Pentacam and Visante, the inferior, nasal, and temporal corneal zones were statistically significantly different (*p* < 0.01). Between Casia and Visante, there were no statistically significant differences in the measured parameter values.

In the control group, statistically significant differences were found for measurements between Pentacam and Casia for the inferior and temporal corneal zones (*p* < 0.01) and nasal corneal zone (*p* = 0.04). Statistically significantly different values were also observed between Pentacam and Visante for the central and inferior corneal zones (*p* < 0.01), nasal corneal zone (*p* = 0.02), and temporal corneal zone (*p* = 0.01). A statistically significant difference was observed between Casia and Visante for only the inferior corneal zone measurement (*p* = 0.03).

**Figure 1:**
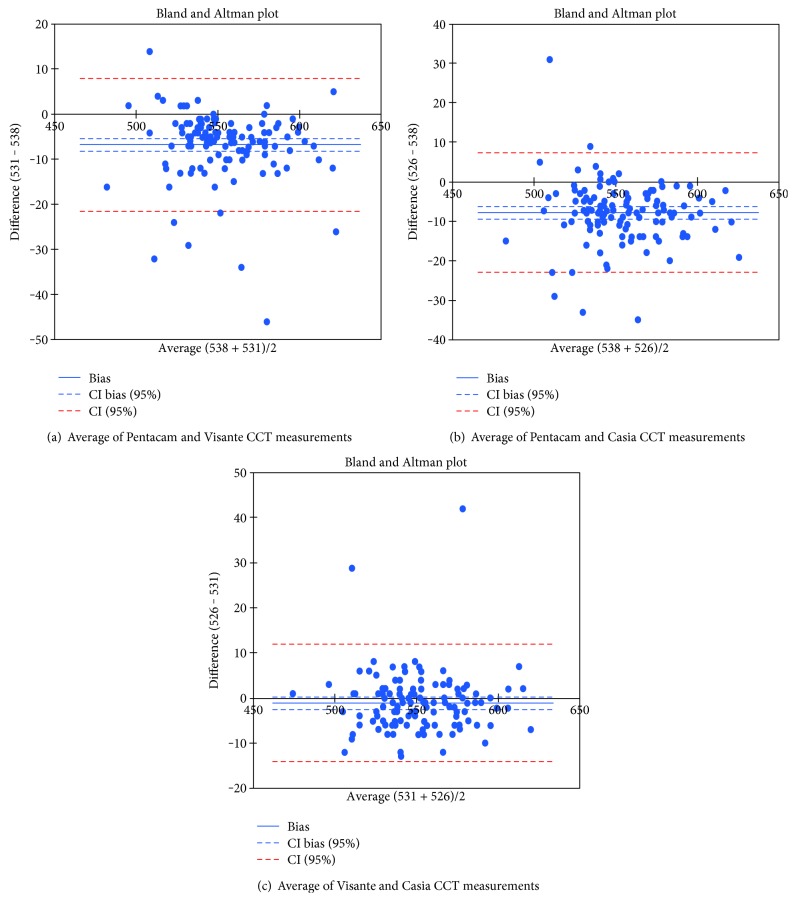
Bland-Altman plots for the agreement between three imaging system measurements in central (CCT—central corneal thickness) and temporal (TCT—temporal corneal thickness) corneal thickness.

Figure 1 shows the Bland-Altman plots for the agreement between three imaging system measurements in central (CCT—central corneal thickness) and temporal (TCT—temporal corneal thickness) corneal thickness. The dotted lines represent mean thickness differences between methods. The interline zones represent the area of 95% limits of agreement.

## 5. Discussion

The results of many studies concerning anterior chamber parameter measurements in eyes with PEX syndrome are different. Not all eyes with PEX syndrome show clinically significant PEX-related keratopathy. Possible explanations may be interindividual differences regarding the involvement of various tissues of the anterior segment in the PEX process. PEX may have been previously misdiagnosed as an atypical non-guttata Fuchs dystrophy [1, 2, 5].

An accurate corneal thickness reading is essential prior to IOP readings, refractive surgery, and many other types of ocular surgeries [5, 13, 15, 18–20]. The mean CCT based on meta-analysis in white adults was 535 *μ*m ± 11.6%. Doughty et al. found that any 10 *μ*m deviation from the mean normal corneal thickness reading results in a 0.5 mmHg difference in measurement when using a Goldmann tonometer [21].

The corneal thickness in eyes with PEX syndrome, which often makes it difficult to manage glaucoma or ocular hypertension, must be assessed precisely to avoid underestimating the IOP readings [2, 10, 19, 20, 22–24].

Reports on CCT in PEX eyes are contradictory. Most studies show that the CCT in patients with PEX syndrome is thinner compared to that in the control non-PEX group [6, 10, 12, 19, 23, 24]. Our findings agree with those reported by Kitsos et al. [22], Tomaszewski et al. [25], and Arnarsson et al. [26]. These differences may result from other sampling methods, ethnic differences, and the number of participants. In our study, the mean CCT in eyes with PEX syndrome as measured using all three instruments was thicker than that in the normal eyes. Although different methods were used for corneal thickness measurement in the PEX group and control non-PEX group, the results of their comparison are coherent.

In our study, we tried to compare the central and peripheral pachymetry measurements by using three different measuring methods and to evaluate the agreement between them. In the reviewed literature, we did not find such a comparison for eyes with diagnosed PEX syndrome. Similar studies were performed for keratoconus [9].

Our results demonstrate the differences in corneal thickness in both central and peripheral zones. In the study group, statistically different measurements were obtained from Pentacam for the temporal, nasal, and inferior peripheral zones. The CCT was statistically different only when measured using Pentacam in comparison with Casia. All three instruments used in this study had high comparability and repeatability for measuring the corneal thickness compared to other studies [11, 14, 16, 18]. The peripheral points measured using Pentacam were overestimated in comparison with those measured using Visante and Casia. These measurements are comparable with the results reported in the previous studies [8, 15, 17]. Better agreement was observed between Visante and Casia measurements [15, 16].

The limitation of our study may be the fact that we did not compare our results with those obtained using ultrasound pachymetry, which is considered the gold standard for CCT measurement. Newer, noncontact methods for measuring corneal thickness are preferred over conventional ultrasonic pachymetry. This results from a patient's discomfort when using the traditional method, risk of transmission of infections, or development of corneal epithelial defects. They offer repeatability and a range of quantitative and qualitative information.

The differences in results are dependent on measurement technique. OCT devices deliver highly comparable results. Scheimpflug camera imaging in many points significantly differs in measurement if compared with Casia or Visante. However, almost equal scan quality and axial resolution do not make this device worse in corneal thickness evaluation in preoperative period or in PEX-related keratopathy evolution analysis.

In conclusion, the results of our study show that the CCT in eyes with PEX syndrome as measured using three different, independent methods is higher than that in the non-PEX group, and despite the variable peripheral corneal thickness, this parameter remains crucial in IOP measurements. This may be a risk factor in the development or progression of glaucoma. However, we must remember about the limitation of axial resolution of all devices. It ranged between 10 and 18 *μ*m. It is below the differences in particular measurement. In long-term observations, CCT data can be applied to evaluate progression of PEX-related keratopathy as well as correction of IOP.

## Figures and Tables

**Table 1:** Mean pachymetry values—PEX group.

Corneal thickness in regions					
Technique	Central	Superior	Inferior	Nasal	Temporal
*Pentacam*	557 ± 28 *μ*m	573 ± 27 *μ*m	562 ± 28 *μ*m	579 ± 25 *μ*m	568 ± 28 *μ*m
*Casia*	549 ± 28 *μ*m	572 ± 29 *μ*m	552 ± 29 *μ*m	564 ± 28 *μ*m	548 ± 28 *μ*m
*Visante*	551 ± 27 *μ*m	565 ± 57 *μ*m	556 ± 27 *μ*m	563 ± 26 *μ*m	552 ± 28 *μ*m

**Table 2:** Mean pachymetry values—non-PEX group.

Corneal thickness in regions					
Technique	Central	Superior	Inferior	Nasal	Temporal
*Pentacam*	552 ± 25 *μ*m	576 ± 22 *μ*m	558 ± 24 *μ*m	576 ± 23 *μ*m	559 ± 24 *μ*m
*Casia*	548 ± 24 *μ*m	570 ± 25 *μ*m	547 ± 23 *μ*m	570 ± 22 *μ*m	547 ± 22 *μ*m
*Visante*	550 ± 24 *μ*m	573 ± 23 *μ*m	554 ± 24 *μ*m	569 ± 22 *μ*m	551 ± 24 *μ*m

**Table 3:** Differences in pachymetry values between PEX and control groups—*p* value.

Region					
Technique	Central	Superior	Inferior	Nasal	Temporal
*Pentacam*	0.15	0.58	0.22	0.37	<0.001
*Casia*	<0.001	0.6	0.19	0.11	0.88
*Visante*	<0.001	0.17	0.67	0.1	0.77
